# NG2 expression in microglial cells affects the expression of neurotrophic and proinflammatory factors by regulating FAK phosphorylation

**DOI:** 10.1038/srep27983

**Published:** 2016-06-16

**Authors:** Lie Zhu, Qing Su, Xiang Jie, Antang Liu, Hui Wang, Beiping He, Hua Jiang

**Affiliations:** 1 Department of Plastic Surgery, Chang Zheng Hospital, Second Military Medical University, Shanghai 200003, China; 2 Department of Ophthalmology, Chang Zheng Hospital, Second Military Medical University, Shanghai 200003, China; 3 Department of Anatomy, Yong Loo Lin School of Medicine, National University of Singapore, Singapore

## Abstract

Neural/glial antigen 2 (NG2), a chondroitin sulfate proteoglycan, is significantly upregulated in a subset of glial cells in the facial motor nucleus (FMN) following CNS injury. NG2 is reported to promote the resulting inflammatory reaction, however, the mechanism by which NG2 mediates these effects is yet to be determined. In this study, we examined the changes in NG2 expressing microglial cells in the FMN in response to facial nerve axotomy (FNA) in mice. Our findings indicated that NG2 expression was progressively induced and upregulated specifically in the ipsilateral facial nucleus following FNA. To further investigate the effects of NG2 expression, *in vivo* studies in NG2-knockout mice and *in vitro* studies in rat microglial cells transfected with NG2 shRNAs were performed. Abolition of NG2 expression both *in vitro* and *in vivo* resulted in increased expression of neurotrophic factors (nerve growth factor and glial derived neurotrophic factor), decreased expression of inflammatory mediators (tumor necrosis factor-α and interleukin-1β) and decreased apoptosis in the ipsilateral facial nucleus in response to FNA. Furthermore, we demonstrated the role of FAK in these NG2-induced effects. Taken together, our findings suggest that NG2 expression mediates inflammatory reactions and neurodegeneration in microglial cells in response to CNS injury, potentially by regulating FAK phosphorylation.

Neural/glial antigen 2 (NG2), also known as chondroitin sulfate proteoglycan 4 (CSPG4), is a member of the chondroitin sulfate proteoglycan family, which comprise integral components of the CNS extracellular matrix. NG2 is constitutively expressed in glia cells, which are ubiquitous throughout the gray and white matter of the developing and mature CNS^1^. NG2 expressing glia cells are a distinct cell type constituting 5–10% of all glial cells found in the normal CNS, which also includes microglia, astrocytes and oligodendrocytes^2^,^3^. These constitutive NG2 expressing cells predominantly display a progenitor phenotype^4^ and respond to injured and diseased neurons by undergoing hypertrophy^5^ or extension of their processes encapsulating damaged neurons^6^,^7^.

Another glial cell type, the microglia, are the first line of defense in the CNS. In the normal brain, microglia exist in a resting state^8^,^9^, however, upon neuronal injury, microglia transform into an activated state where they translocate to, and adhere to, damaged neurons. If cell death occurs, microglia acquire phagocytic function removing neuronal cell debris^10^,^11^. A rapid increase in NG2 expression has been proposed to be a feature of CNS injury^12^,^13^. Our previous study^14^ indicated that axotomized facial motor nucleus (FMN) showed two types of NG2 expressing cells, constitutive NG2 cells and induced NG2 expressing microglial cells. We showed that in response to facial nerve axotomy (FNA) in the adult rat, activated microglia were induced to express NG2. The induction of NG2 expression in activated microglial cells leads to drastic changes in morphology and function, with wide ranging effects on the production of a variety of inflammatory mediators^15^, including proinflammatory cytokines, chemokines and reactive oxygen species, along with the release of nitric oxide^16^,^17^. The secretion of neuroprotective factors^18^ and the loss of phagocytic capability^19^ have also been reported in response to the induction of NG2 expression in activated microglial cells.

Focal adhesion kinase (FAK) plays a central role in the macromolecular assembly of focal adhesions, integrin-mediated protein complexes that link the actin cytoskeleton to the extracellular matrix^20^. Activated FAK mediates several cellular signaling pathways involved in cell adhesion, growth, proliferation, migration, survival and angiogenesis^21^. The dysregulation of FAK activity has been linked to tumorigenesis and metastasis^22^. A link between the transmembrane proteoglycan NG2 receptor and FAK has been reported with NG2 regulating FAK phosphorylation via cellular signaling^23^.

We previously demonstrated that NG2 expressing glia cells and microglial cells were activated in axotomized FMN^14^, a well-established model system in which the permeability of the blood-brain barrier is minimally effected, and also in the cortex after lipopolysaccharide (LPS) focal injection into the rat brain^19^,^24^. In this study, we investigate the role of NG2 in the expression of neurotrophic factors and inflammatory mediators in activated microglial cells. Furthermore, we investigate the mechanisms by which NG2 mediates these effects and demonstrate the role of FAK.

## Results

### Increased NG2 expression in the ipsilateral facial nucleus (FN) of mice exposed to FNA

**Figure 1:**
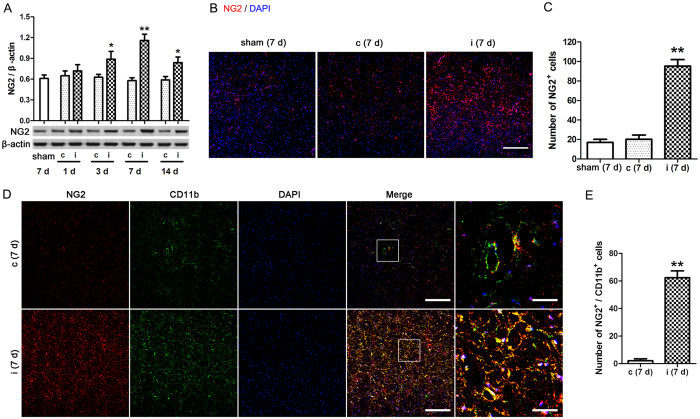
NG2 expression in the facial nucleus (FN) of mice exposed to facial nerve axotomy (FNA). Wild type mice were exposed to FNA and the contralateral (c) and the ipsilateral (i) FN were separately collected at 1, 3, 7, and 14 days. The right FN of the sham operated mice at 7 days served as a control (sham). (**A**) NG2 protein expression levels in the FN were measured by western blot analysis. The relative protein levels were determined after normalization to β-actin expression levels. *p < 0.05, **p < 0.01 vs. sham. (**B**) Immunofluorescence labelling of NG2 (red) in the FN from day 7, nuclei were stained with DAPI (blue). Scale bar = 100 μm. (**C**) From the immunofluorescent images, the number of NG2-expressing cells in the FN from day 7 were counted. Average counting area: 0.1 mm^2^, **p < 0.01 vs. sham. (**D**) Double immunohistochemical staining of FN sections from day 7 combining NG2 (red) with CD11b (green) labeling. Nuclei were stained with DAPI (blue). Scale bar = 100 μm or 20 μm. (**E**) From the stained sections, the number of NG2 and CD11b double labeled cells in the FN from day 7 were counted. Average counting area: 0.1 mm^2^, **p < 0.01. n = 3 mice used in the sham group, n = 4–6 mice used in the FNA group per time point.

Wild type mice were exposed to FNA and the contralateral and the ipsilateral FN were separately collected at 1, 3, 7, and 14 days. Then, NG2 protein expression levels in the FN were measured by western blot analysis (Fig. 1A). No significant differences were observed in NG2 expression in the contralateral FN following FNA. However, in the ipsilateral FN NG2 expression levels increased, with the highest expression observed 7 days after FNA (p < 0.01 compared with sham mice). Next, FN sections from day 7 were subjected to immunohistochemical staining using a fluorescent-tagged mouse anti-NG2 antibody (red), and nuclei were stained with DAPI (blue) (Fig. 1B). Higher expression of NG2 was evident in the ipsilateral FN compared with the contralateral FN. On enumeration of the cells, significantly higher numbers of NG2-expressing cells were detected in the ipsilateral FN compared with the contralateral FN from day 7 (p < 0.01 compared with sham mice) (Fig. 1C). Double immunohistochemical staining of FN sections from day 7 combining NG2 labeling (red) with CD11b labeling (green), a marker of microglia/macrophage, indicated colocalization of these markers in ipsilateral FN (Fig. 1D). On enumeration, significantly higher numbers of NG2 and CD11b double labeled cells were detected in the ipsilateral FN compared with the contralateral FN from day 7 (p < 0.01; Fig. 1E). These findings indicated that NG2 expression is induced specifically in the ipsilateral FN following FNA, with NG2 levels peaking around 7 days.

### Increased expression of neurotrophic factors, decreased expression of inflammatory mediators and decreased apoptosis in NG2-knockout mice

**Figure 2:**
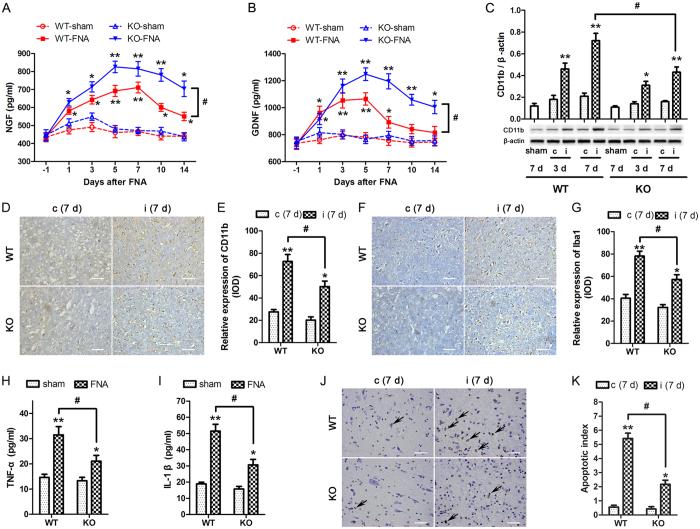
Analysis of the blood and facial nucleus (FN) of wild type and NG2-knockout mice following facial nerve axotomy (FNA). Wild type (WT) or NG2-knockout (KO) mice were exposed to FNA and blood samples and the FN were collected the day before the operation (−1) or at the indicated times after FNA. Serum levels of NGF (**A**) and GDNF (**B**) were measured by ELISA. *p < 0.05, **p < 0.01 vs. corresponding WT or KO sham group. ^#^p < 0.05. (**C**) CD11b expression in the contralateral (c) and ipsilateral (i) FN was measured by western blotting. The sham mice served as control. The relative protein levels were determined after normalization with β-actin. *p < 0.05, **p < 0.01 vs. sham of WT or KO. ^#^p < 0.05. (**D–G**) The contralateral (c) and the ipsilateral (i) facial nucleus (FN) were collected on day 7 after FNA. Immunochemical analysis (IHC) of CD11b (**D**) and Iba1 (**F**) in FN. The CD11b (**E**) and Iba1 (**G**) expression intensities were analyzed in terms of integral optical density (IOD). *p < 0.05, **p < 0.01 vs. corresponding WT or KO c (7 d) group. ^#^p < 0.05. Blood samples were collected at 7 day after FNA, serum levels of TNF-α (H) and IL-1β (**I**) were measured by ELISA. *p < 0.05, **p < 0.01 vs. corresponding WT or KO sham group. ^#^p < 0.05. (**J,K**) The contralateral (c) and the ipsilateral (i) facial nucleus (FN) were collected at 7 day after FNA. (**J**) TUNEL assay was performed on the FN sections from each group. Scale bar = 20 μm. (**K**) The apoptotic index was derived from TUNEL staining of the FN from each group. *p < 0.05, **p < 0.01 vs. corresponding WT or KO c (7 d) group. ^#^p < 0.05. n = 3 in sham group, n = 4–6 in FNA group per time point.

Next, the effects of NG2 expression were analyzed in the blood and FN of WT and NG2 KO mice following FNA. Before these experiments were performed, the effects of ablation of NG2 on the motor functions of the mice were assessed by rotarod and Catwalk analysis (as described in the Supplementary Information). No significant differences were observed between WT and NG2 KO mice in these tests (Fig. S1). Blood samples and the FN were collected the day before the operation (−1) or between 1–14 days after FNA. The serum levels of the neurotrophic factors, nerve growth factor (NGF) and glial derived neurotrophic factor (GDNF), were first measured by ELISA (Fig. 2A,B). FNA induced a significant increase in expression of both NGF and GDNF in WT and KO mice, generally peaking around day 5 (p < 0.01 vs. corresponding WT or KO sham group). However, the expression levels of both of these neurotrophic factors were higher in KO mice compared with WT mice (p < 0.05), indicating the suppression of these factors by NG2. Next, CD11b expression in the contralateral and ipsilateral FN after FNA was measured for the WT and KO mice by western blotting (Fig. 2C). CD11b expression was unchanged in the contralateral (c) FN of WT and KO mice, however in the ipsilateral FN, CD11b expression was increased in both the WT and KO mice. At 7 days after FNA, CD11b expression was significantly higher in the WT mice compared with the KO mice (p < 0.05) indicating that NG2 triggered a shift towards CD11b expressing phagocytic cells. Immunohistochemical analysis of CD11b (Fig. 2D,E) and Iba1 (Fig. 2F,G) expression confirmed these findings indicating significantly higher expression in the WT mice compared with the KO mice in the ipsilateral FN (p < 0.05) following FNA.

To assess the effect of NG2 on the expression of inflammatory mediators, the serum levels of TNF-α and IL-1β were measured from blood samples collected on day 7 after FNA by ELISA (Fig. 2H,I). The expression levels of both factors were increased in response to FNA, but the serum levels were significantly higher in the WT compared with the KO mice (p < 0.05) indicating the stimulatory effect of NG2 on the expression of inflammatory mediators. To assess the potential effect of NG2 on apoptosis, a TUNEL assay was performed on sections of the contralateral and ipsilateral FN collected on day 7 after FNA for the WT and KO mice (Fig. 2J). The apoptotic index values derived from TUNEL staining of the FN indicated that FNA induced apoptosis specifically in the ipsilateral FN (Fig. 2K). Furthermore, the proportion of apoptotic cells was significantly higher in the FN of WT mice compared with KO mice (p < 0.05) indicating the role of NG2 in promoting apoptosis in the ipsilateral FN in response to FNA.

### Role of FAK in the NG2-induced expression of inflammatory mediators and suppression of neurotrophic factors

**Figure 3:**
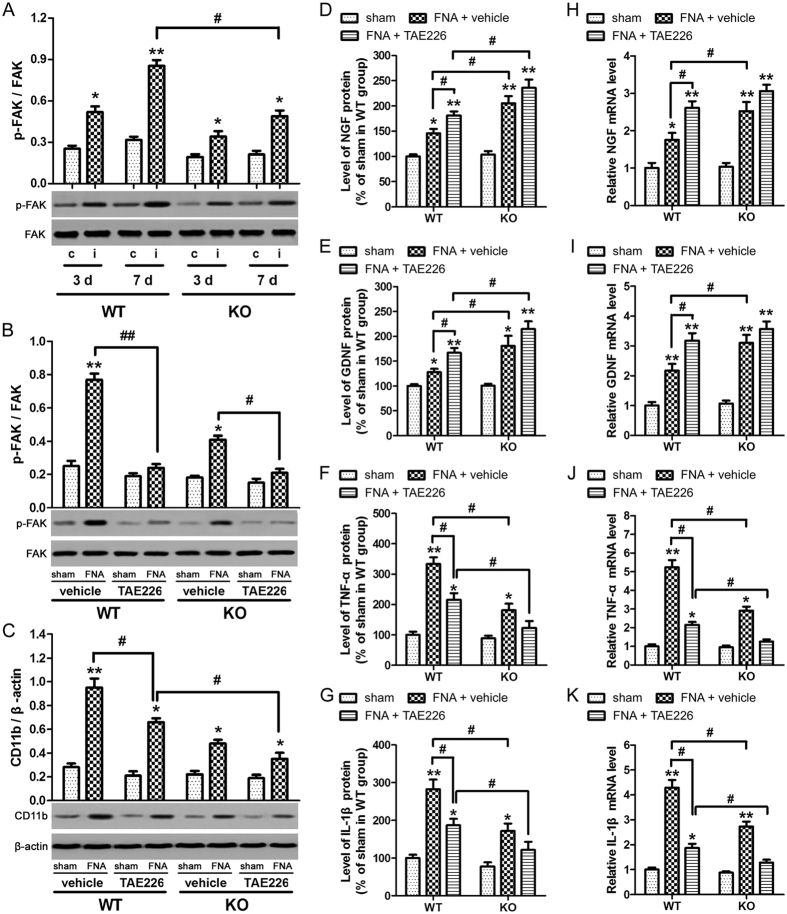
Role of FAK in the expression of neurotrophic factors and inflammatory mediators in wild type and NG2-knockout mice after facial nerve axotomy (FNA). (**A**) Wild type (WT) or NG2-knockout (KO) mice were exposed to FNA. The contralateral (c) and ipsilateral (i) facial nucleus (FN) were collected at 3 or 7 days after FNA, and p-FAK and FAK expression levels in the FN were measured by western blotting. The ratios of phosphorylated to total FAK were quantified by densitometric analysis. *p < 0.05, **p < 0.01 vs. corresponding (c) group at 3 d or 7 d. ^#^p < 0.05. (**B**–**K**) WT or KO mice were exposed to FNA. FAK inhibitor TAE226 (30 mg/kg) or methylcellulose as a vehicle control were orally administered once a day after FNA and continued for 7 days. Ipsilateral FN or blood samples were collected 7 days after FNA. p-FAK (**B**) and CD11b (**C**) in the FN were measured by western blotting. The sham mice served as control (sham). Results of densitometric analysis were normalized to total FAK or β-actin. *p < 0.05, **p < 0.01 vs. sham of each group. ^#^p < 0.05. Serum levels of NGF (**D**), GDNF (**E**), TNF-α (**F**) and IL-1β (**G**) were measured by ELISA. Data were expressed as a percentage of the levels for the sham in the WT mice group (NGF, 100% = 421.58 ± 35.72 pg/mL; GDNF, 100% = 711.24 ± 48.34 pg/mL; TNF-α, 100% = 12.51 ± 1.63 pg/mL; IL-1β, 100% = 16.45 ± 1.84 pg/mL). NGF (**H**), GDNF (**I**), TNF-α (**J**) and IL-1β (**K**) mRNA expression of ipsilateral FN as determined by qRT-PCR. Data were normalized to GAPDH. *p < 0.05, **p < 0.01 vs. sham of each group. ^#^p < 0.05. n = 3 in sham group, n = 4–6 in FNA.

To investigate the role of FAK in the expression of neurotrophic factors and inflammatory mediators in WT and NG2 KO mice, p-FAK and FAK levels in the contralateral and ipsilateral FN from days 3 and 7 after FNA, were determined by western blotting (Fig. 3A). The ratios of phosphorylated to total FAK were quantified by densitometric analysis and were found to be unchanged in the contralateral FN. However, in the ipsilateral FN, the ratio of active FAK was significantly increased in the WT mice compared with KO mice on day 7 after FNA (p < 0.05). To investigate further, FAK inhibitor TAE226 was employed. Prior to these experiments, chemical analysis of the plasma isolated from WT and KO mice treated with or without FAK inhibitor was performed (as detailed in the Supplementary Information) to ensure that this inhibitor did not promote any unwanted side effects in the mice. However, no significant chemical changes were detected in response to treatment with FAK inhibitor (Table S1). FAK inhibitor TAE226 (30 mg/kg) or methylcellulose (used as a vehicle control) were orally administered once a day after FNA in WT and KO mice and continued for 7 days, at which point ipsilateral FN and blood samples were collected. Analysis of p-FAK and CD11b in the ipsilateral FN were measured by western blotting (Fig. 3B,C). TAE226 significantly attenuated p-FAK expression in WT and KO mice. The effect of this FAK inhibitor was then analyzed on CD11b expression and found to significantly increase CD11b expression in WT mice with or without exposure to FNA compared with the vehicle alone (p < 0.05). A significant reduction in CD11b expression levels was observed between FNA-exposed TAE226-treated WT and KO mice (p < 0.05), indicating that NG2 induces CD11b expression only in the absence of phosphorylated FAK. Next, the role of FAK in the NG2-regulated expression of inflammatory mediators and neurotrophic factors after FNA was investigated by ELISA. The serum levels of NGF (Fig. 3D) and GDNF (Fig. 3E) showed a significant increase in the NG2 KO mice compared with the WT mice (p < 0.05), indicating NG2-induced suppression of these neurotrophic factors in WT mice. This effect was also seen in the presence of TAE226. By contrast, the serum levels of TNF-α (Fig. 3F) and IL-1β (Fig. 3G) showed a significant decrease between WT and KO mice (p < 0.05), indicating that NG2 promoted the expression of these inflammatory mediators in WT mice. This effect was evident in the presence or absence of the FAK inhibitor, TAE226. Finally, the mRNA expression levels of these neurotrophic factors and inflammatory mediators were assessed by qRT-PCR (Fig. 3H–K), confirming that the RNA levels reflected the protein levels. Taken together, these findings demonstrated that NG2 suppresses the expression of neurotrophic factors but promotes the expression of inflammatory mediators after FNA in mice microglial cells. However, unlike our *in vitro* findings, the NG2-induced suppression of neurotrophic factors was not abolished in the presence of FAK inhibitor indicating that FAK may not be involved mechanistically *in vivo*.

### NG2 expression in microglial cells in response to LPS-induced pathology

**Figure 4:**
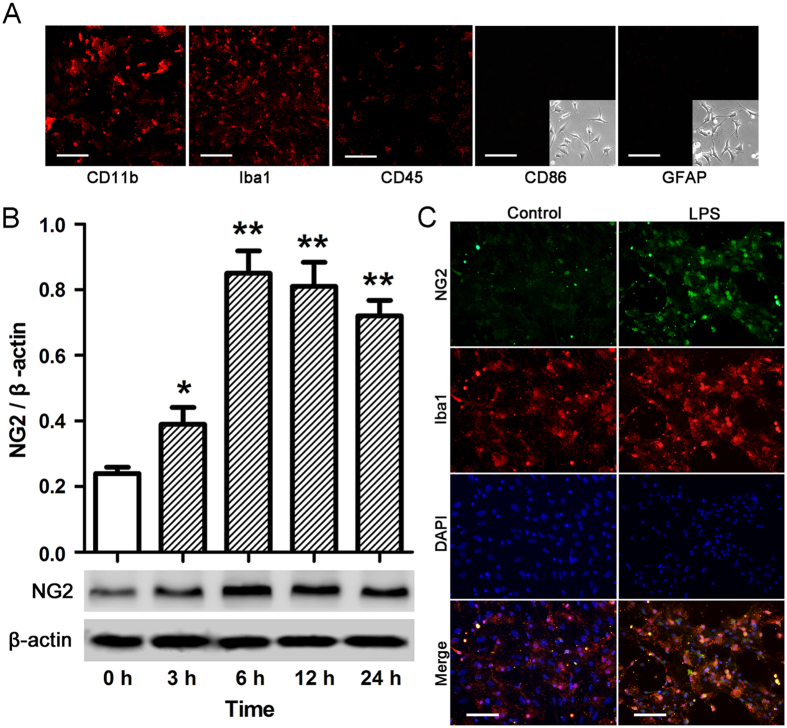
NG2 expression in microglial cells treated with or without LPS. (**A**) Microglial cells were stained for CD11b, Iba1, CD45, CD86 (insert, phase contrast) and GFAP (insert, phase contrast) to assess the purity of the culture. Scale bar = 50 μm. (**B**) Microglial cells were treated with 10 ng/ml LPS for the indicated times, and NG2 expression was measured by western blotting. The relative protein levels were determined after normalization with β-actin. *p < 0.05, **p < 0.01 vs. 0 h. (**C**) Microglial cells, treated with 10 ng/ml LPS for 6 h, were subjected to double immunohistochemical staining combining NG2 (green) with Iba1 (red) labeling. Nuclei were stained with DAPI (blue). Untreated microglial cells cultured for 6 h served as a control. Scale bar = 25 μm. Data are represented as means ± s.d. (n = 3, technical replicates). Each experiment was repeated on at least two separate occasions.

Primary cortical microglial cultures were prepared from the cerebral hemispheres of 1–2 day old postpartum male Sprague–Dawley rats and the purity of these cultures was confirmed by histochemical detection using mouse anti-CD11b, rabbit anti-Ibal, mouse anti-CD45, mouse anti-CD86, and mouse anti-GFAP antibodies, as shown in Fig. 4A. Cultures that showed greater than 96% purity were used for further experiments. To analyze the effect of LPS-induced pathology on NG2 expression in microglial cells, cells were treated with 10 ng/ml LPS for the indicated times, and NG2 expression was measured by western blotting (Fig. 4B). NG2 expression levels significantly increased in microglial cells in response to LPS treatment, peaking at 6 h (p < 0.01 compared with 0 h). Double immunohistochemical staining of LPS-treated (10 ng/mL for 6 h) microglial cells with Iba1 (green), to identify the cells, and NG2 (red) revealed induction of NG2 expression in microglial cells exposed to LPS compared with barely detectable NG2 immunoreactivity in untreated microglial cells (Fig. 4C). These findings indicated that NG2 was expressed in response to LPS in microglial cells.

### Inhibition of NG2-induced effects in microglial cells by transfection with NG2-specific shRNA

**Figure 5:**
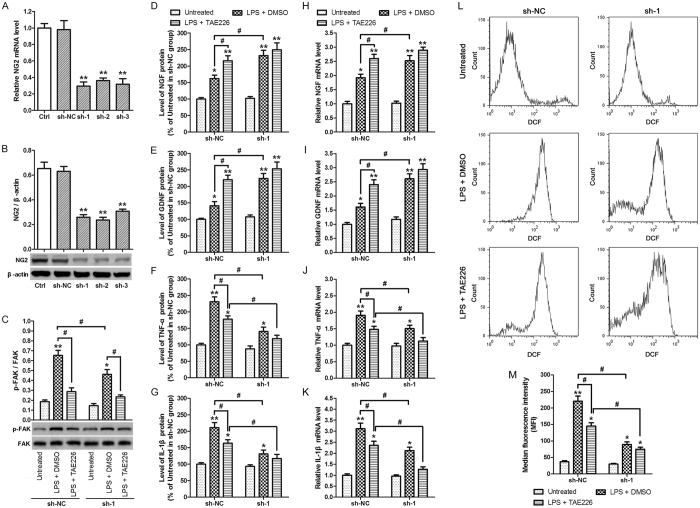
Analysis of NG2 function using NG2 shRNA. Microglial cells were transfected with control shRNA (sh-NC), NG2 shRNA1 (sh-1), NG2 shRNA2 (sh-2) or NG2 shRNA3 (sh-3) for 48 h. (**A**) NG2 mRNA expression as determined by qRT-PCR. Data were normalized to GAPDH. **p < 0.01 vs. non-transfected control cells (Ctrl). (**B**) NG2 protein expression as determined by western blot analysis. Data were normalized to β-actin. **p < 0.01 vs. non-transfected control cells (Ctrl). (**C**–**M**) Cells transfected with control shRNA (sh-NC) or NG2 shRNA1 (sh-1) were pretreated with either 10 μM of TAE226 or DMSO for 2 h, and then treated with 10 ng/ml LPS for 6 h. (**C**) p-FAK and FAK expression as determined by western blotting. The ratios of phosphorylated to total FAK were quantified by densitometric analysis. NGF (**D**), GDNF (**E**), TNF-α (**F**) and IL-1β (**G**) levels in the culture medium were measured by ELISA. Data were expressed as percentage of levels in untreated of sh-NC group (NGF, 100% = 9.16 ± 1.02 pg/mL; GDNF, 100% = 42.02 ± 5.19 pg/mL; TNF-α, 100% = 60.75 ± 8.31 pg/mL; IL-1β, 100% = 81.29 ± 10.57 pg/mL). NGF (**H**), GDNF (**I**), TNF-α (**J**) and IL-1β (**K**) mRNA expression of microglial cells as determined by qRT-PCR. Data were normalized to GAPDH. (**L**) Intracellular ROS levels determined by flow cytometry using DCFH-DA staining. (**M**) The intracellular ROS data were quantified by the median fluorescence intensity (MFI). *p < 0.05, **p < 0.01 vs. untreated group. ^#^p < 0.05. Data are represented as the mean ± s.d. (n = 3, technical replicates). Each experiment was repeated on at least two separate occasions.

To investigate the effects of NG2 in microglial cells, cells were transfected with control shRNA (sh-NC), NG2 shRNA1 (sh-1), NG2 shRNA2 (sh-2) or NG2 shRNA3 (sh-3) for 48 h, and NG2 mRNA expression was determined by qRT-PCR (Fig. 5A). The mRNA expression of NG2 was significantly reduced in sh-1, sh-2 and sh-3 compared with non-transfected control cells (p < 0.01). Similarly, NG2 protein expression was significantly reduced in sh-1, sh-2 and sh-3 compared with non-transfected control cells (p < 0.01) as determined by western blot analysis (Fig. 5B). Next, the expression of p-FAK and FAK was measured by western blotting in normal microglial cells (Ctrl) or cells transfected with NG2 shRNA1 (sh-1) that had been pretreated with either 10 μM of TAE226 or DMSO for 2 h, and then treated with 10 ng/mL LPS for 6 h (Fig. 5C). A significant difference was evident in pFAK expression between the control cells and sh-1-transfected cells treated with LPS alone, and this difference was abolished on treatment with the FAK inhibitor TAE226. Finally, the role of NG2 in the expression of inflammatory mediators and neurotrophic factors in microglial cells after LPS treatment was investigated by ELISA of the culture medium (Fig. 5D–G). The levels of NGF (Fig. 5D) and GDNF (Fig. 5E) in the culture medium showed a significant increase in sh-1-transfected cells compared with non-transfected control cells after LPS treatment (p < 0.05) and this increase was abolished in the presence of TAE226 indicating that FAK is required for the NG2-induced suppression of neurotrophic factors in microglial cells after LPS treatment. By contrast, the levels of TNF-α (Fig. 5F) and IL-1β (Fig. 5G) showed a significant decrease in expression between non-transfected control cells and sh-1-treated cells after LPS treatment (p < 0.05) and this decrease was not abolished in the presence of TAE226 indicating that FAK may not be required for the NG2-induced promotion of inflammatory mediators in microglial cells after LPS treatment. The mRNA expression levels of these neurotrophic factors and inflammatory mediators were also assessed by qRT-PCR (Fig. 5H–K), confirming that the RNA levels reflected the protein levels. Finally, cells transfected with sh-NC or sh-1, pretreated with either TAE226 or DMSO, then treated with LPS, were subjected to DCFH-DA staining and flow cytometry to determine intracellular reactive oxygen species (ROS) levels (Fig. 5L). The intracellular ROS data were quantified by determining the median fluorescence intensity (MFI) values (Fig. 5M). It is clear that in LPS-stimulated cells, intracellular ROS levels are significantly reduced in response to silencing of NG2 (p < 0.05). NG2 expression has previously been linked to the generation of free radicals and it is well established that free radicals can perpetuate an inflammatory response. Our findings therefore confirm that NG2 plays a role in elevating intracellular ROS levels and therefore triggering an inflammatory response.

Taken together, these findings suggest that NG2 suppresses the expression of neurotrophic factors but promotes the expression of inflammatory mediators in microglial cells after LPS treatment, and that the former is FAK-dependent.

## Discussion

In this study, we examined the changes in NG2-expressing microglial cells in the FMN in response to facial nerve transection in mice. It has previously been established that NG2 is significantly upregulated in a subset of glial cells in response to facial nerve transection in an animal model resulting in hypertrophy of NG2-expressing cells and NG2 encapsulation of axotomized motoneurons, effectively deactivating the damaged motoneurons by separating them from presynaptic terminal boutons^14^. Dawson *et al*.^25^ showed that NG2 expressing cells in normal adult CNS do not express the same set of markers known to be specific for other glial cell types, confirming that this subset of encapsulating NG2-expressing glial cells were not astrocytes, oligodendrocytes or resting microglia but in fact were activated microglial cells.

Increased expression of NG2 is a characteristic feature of CNS injury^12^,^13^. Our findings indicated that NG2 expression was induced specifically in the ipsilateral FN following FNA, with NG2 levels peaking around 7 days after nerve injury. This confirmed previous findings^14^ suggesting that NG2 protein expression may be progressively induced and upregulated in response to FNA. A similar NG2 expression profile was observed in our study *in vitro* in rat microglial cells following exposure to LPS. Similarly, increased NG2 expression in the brain cortex has been reported after LPS-induced focal injury^26^. Gao *et al*. demonstrated that injection of rats with LPS promoted the expression of NG2 and, *in vitro*, LPS induced the expression of NG2 in primary microglial cells. It has also been reported that LPS induces the expression of iNOS and proinflammatory cytokines in microglia in a similar way to FNA^15^. We therefore used LPS stimulation in this study to induce this type of inflammatory response.

It is well established that the activation of microglial cells in a variety of neurodegenerative diseases enhances the release of proinflammatory cytokines TNF-α and IL-1 and induces the expression of inducible nitric oxide synthase, which in turn catalyzes nitric oxide production^27^,^28^. These factors may contribute to neurodegeneration in neurological diseases^29^. In the present study, knockout of NG2 in mice resulted in increased expression of neurotrophic factors (NGF and GDNF), decreased expression of inflammatory mediators (TNF-α and IL-1β), elevated levels of intracellular ROS and decreased apoptosis in the ipsilateral FN in response to FNA. The potential neuroprotective role of NG2 has previously been proposed^18^,^30^, however, this is likely offset by the role of NG2 in mediating an inflammatory reaction.

In the present study, we investigated the mechanistic link between NG2 and proinflammatory cytokines. As well as its role in the downstream signaling of integrins, FAK is proposed to be a mediator of the inflammatory response through the activation of mitogen-activated protein kinases. We demonstrated the putative role of FAK in the NG2-induced expression of inflammatory mediators, the suppression of neurotrophic factors and the induction of apoptosis. Activation of FAK has been reported to elicit intracellular signal transduction pathways that promote cell contact and adhesion to the extracellular matrix, playing a role in cell migration^31^. FAK has also been linked with cell proliferation, angiogenesis and apoptotic cell death^21^. In response to facial nerve injury, our findings suggest that NG2 expression in microglial cells affects the expression of neurotrophic and proinflammatory factors, potentially by regulating FAK phosphorylation. TNF-α has been shown to dose-dependently induce the phosphorylation of FAK in human periodontal ligament fibroblasts^32^, and in their study knockdown of FAK decreased the production of TNF-α-induced interleukins (IL-6 and IL-8). FAK phosphorylation has previously been shown to be regulated by signaling via the transmembrane proteoglycan NG2 receptor^23^. Based on our *in vitro* findings, we therefore propose that NG2 may mediate its proinflammatory effects via phosphorylation of FAK, and that the increased levels of inflammatory cytokines, such as TNF-α, may perpetuate and amplify the inflammation by in turn activating more FAK. The mechanism by which NG2 suppresses neurotrophic factors is yet to be determined, but also appears from our findings to involve FAK. Further studies are required to characterize how FAK signaling acts downstream of these inflammatory cytokines to modulate the expression of neurotrophic factors and our results need to be confirmed *in vivo*. Our findings also indicated a potential role for FAK in the apoptotic process. Proteoglycans and FAK have previously been implicated in the regulation of the apoptotic pathway^33^,^34^ although the mechanisms remained unclear.

Our findings shed new light on the role of activated microglia in neuropathogenesis and indicate the potential involvement of FAK in NG2-mediated inflammatory reactions and neurodegeneration in response to CNS injury, although further studies are required. The mechanistic details also require further investigation and the direct role of FAK is yet to be confirmed. Furthermore, NG2 is important in the development of the oligodendrocyte lineage and Kucharova & Stallcu^35^ reported that ablation of NG2 resulted in delayed expansion of the pool of oligodendrocyte progenitor cells, with a subsequent delay in oligodendrocyte production and the development of myelinating processes. The wider implications of NG2 ablation therefore should also be considered. However, the downregulation of NG2 in activated microglia could be a potential future therapeutic strategy for inflammation-induced neurodegenerative diseases.

## Materials and Methods

### Reagents

A stock solution of the FAK inhibitor TAE226 (Novartis Phamaceuticals, East Hanover, NJ, USA) was reconstituted with dimethyl sulfoxide (DMSO; Sigma, St. Louis, MO, USA) and diluted with culture media before use. The final DMSO concentration in all cultures was 0.025%. For mouse experiments, TAE226 was dissolved in 0.5% methylcellulose to a final concentration of 10 mg/ml and administered orally.

### Animal model of facial nerve axotomy (FNA)

Wild-type (WT) and NG2-knockout (KO) C57BL/6J littermate male mice, 3–5 months-of-age, were purchased from The Jackson Laboratory (Bar Harbor, ME, USA). Mice were exposed to FNA and the contralateral and the ipsilateral facial nucleus (FN) were separately collected over a 14-day period after FNA. All experiments were performed in accordance with the Chinese legislation on the use and care of laboratory animals and were approved by the Institutional Animal Care and Use Committee of Chang Zheng Hospital. The animals were maintained in a 12-h day/night cycle and provided with food and water *ad libitum*.

The mice were then subjected to FNA performed according to a previously published surgical procedure^14^. Briefly, the mice were anesthetized with xylazine (80 mg/kg) and ketamine (10 mg/kg). Under a dissecting microscope, the left facial nerve was exposed at its exit from the stylomastoid foramen and transected. To disconnect the distal and proximal ends of the left facial nerve, a segment of approximately 5 mm of the distal stump was removed. The right facial nerve was not operated upon. Sham operated controls were included in which only a skin incision was made exposing the FN. The FN contralateral and ipsilateral to the axotomy in the FNA operated mice or the right FN for the sham operated controls were separately collected at 1, 3, 7, and 14 days. Three to six animals at each time point were anesthetized and then perfused via the left ventricle with ice-cold Ringer’s solution (pH 7.4) and 2% paraformaldehyde (pH 7.4). The brainstem with the cerebellum was removed, fixed in the same solution for 4 h at 4 °C, then immersed in 20% sucrose in 0.1 M phosphate-buffered saline (PBS) overnight at 4 °C. The frozen tissue was sectioned (20–30 μm thickness) using a cryostat (Leica CM 3050, Bensheim, Germany).

### Experimental design

Wild type (WT) and NG2-knockout (KO) mice were used in the experiments and were treated as sham controls (n = 3 per time point) or exposed to FNA (n = 6 per time point). Blood samples and the contralateral (c) or ipsilateral (i) facial nucleus (FN) were collected the day before the operation (−1) or at the indicated times after FNA.

FAK inhibitor experiments included WT and KO mice exposed to FNA (n = 6 for each group) and the respective sham controls (n = 3 for each group). FAK inhibitor TAE226 (30 mg/kg) or methylcellulose, as a vehicle only control, were orally administered once a day after FNA for 7 days. Ipsilateral FN or blood samples were collected 7 days after FNA.

### Microglial cell culture

Primary cortical microglial cultures were prepared from the cerebral hemispheres of 1–2 day old postpartum male Sprague–Dawley rats according to a previously reported method^36^, with some modifications. The rats were purchased from the Shanghai Laboratory Animal Center of the Chinese Academy of Sciences (Shanghai, China). Dissociated cells (1 × 10^6^ cells/mL) were seeded in Dulbecco’s modified Eagle’s medium (DMEM)/F12 (Invitrogen, Carlsbad, CA, USA) supplemented with 10% fetal bovine serum (Hyclone, Logan, UT, USA), 1 × antibiotic-antimycotic (Sigma), 10 μg/ml insulin (Sigma) (complete medium) and incubated at 37 °C in a humidified atmosphere of 95% air and 5% CO_2_. After 24 h, the culture medium was replaced and this was repeated every 3–4 days. After 2 weeks, confluent cells were purified by a mild trypsinization method^37^. Briefly, confluent microglial cells were treated with trypsin solution (Invitrogen; 0.25% trypsin and 1 mM EDTA, diluted 1:3 in DMEM) for 10 min at 37 °C. Nonmicroglial cells detached as a single layer, whereas microglial cells remained attached to the bottom of the flask. The medium containing the layer of detached cells was replaced with fresh conditioned medium for 24 h. The purity of the microglia was assessed by histochemical detection using mouse anti-CD11b (1:100; Santa Cruz Biotechnology, Santa Cruz, CA, USA), rabbit anti-Ibal (1:100; Abcam, Cambridge, MA, USA), mouse anti-CD45 (1:100; Abcam), mouse anti-CD86 (1:100; Santa Cruz Biotechnology) and mouse anti-GFAP (1:100; Abcam), and cultures with above 96% purity were used for further experiments.

### Knockdown of NG2 in microglia cells using short-hairpin RNAs (shRNAs)

For knockdown of NG2, three shRNA oligonucleotide sequences were designed to the NG2 sequence: shRNA1, CCCTATCTTCACGTAGCCAAT; shRNA2, GCAACCAACTTGTGGAACATT; and shRNA3, CCGAACAGAATCTAGGAGCAA. A scrambled sequence, shRNA-NC, CAAGGACATGCGTCAGAGGGT, was also designed as a control. Sequence homologies were checked using NCBI BLAST searches (http://blast.ncbi.nlm.nih.gov/Blast.cgi). These three NG2 shRNA sequences and the scrambled control sequence were cloned into the lentiviral expression vector pLVTHM (System Biosciences, SBI, Mountain View, CA, USA) at the *Bam*HI/*Mlu*I site to give pLVTHM shNG2. Lenitviral vectors were produced using standard techniques by transient transfection of HeLa cells as described previously^38^. Virus was harvested 48 h post-transfection and concentrated by ultracentrifugation at 27 000 × *g* for 2.5 h. Virus was then aliquoted and stored at −80 °C.

Microglial cells (1 × 10^5^), resuspended in DMEM/F12 medium, were transduced with pLVTHM containing control shRNA (sh-NC), NG2 shRNA1 (sh-1), NG2 shRNA2 (sh-2) or NG2 shRNA3 (sh-3) at a multiplicity of infection of 100 for 48 h. Quantitative PCR was performed on the cDNA to assess the effectiveness of NG2 knockdown. GAPDH was used as a control gene.

### Immunohistochemistry

For immunofluorescent staining, FN sections or microglial cells, treated with 10 ng/ml LPS for 6 h, were washed with 1 × PBS plus 0.1% Triton X-100 for 10 min three times and then blocked with 5% normal goat serum for 1 h at room temperature. Next, the sections or cells were incubated with mouse anti-NG2 antibody (1:500; Chemicon, Temecula, CA, USA) and/or anti-CD11b (1:100; Santa Cruz Biotechnology) or anti-Iba1 (1:100; Abcam) overnight at room temperature. Following another three washes 1 × PBS, the sections or cells were incubated with goat anti-mouse Alexa Fluor 488 (Invitrogen) for 1 h. DAPI solution was then added for 2 min to stain the nuclei. Finally, sections or cells were mounted onto microscope slides using nonfluorescent mounting medium (Dako, Glostrup, Denmark) and were visualized using a Laser-Scanning Confocal Microscope (Olympus FluoView™ FV1000, Tokyo, Japan). Five randomly selected microscopic fields were analyzed blind per slide and the number of NG2 and/or CD11b or Iba1 positive cells was recorded.

### Western blot analysis

Wild type mice were exposed to FNA and the contralateral and the ipsilateral FN were separately collected after FNA exposure. Total proteins were extracted from the FN tissue using M-PER tissue Protein Extraction Reagent (Pierce, Rockford, IL, USA), EDTA, and protease inhibitor cocktail (Pierce). Alternatively, microglial cells were treated with 10 ng/ml LPS for the indicated times and then cells were harvested and lysed in cell lysis buffer for 30 min on ice along with protease inhibitor cocktail (Pierce). The concentration of total protein from FN tissue or microglial cells was measured according to Bradford’s method^39^ and equivalent amounts of protein were loaded onto gels and separated by SDS-PAGE. Proteins were transferred to PVDF membrane (Bio-Rad Laboratories, Hercules, CA, USA) and membranes were blocked for 1 h with 5% milk in TBS-0.1% Tween buffer. Membranes were then incubated with primary antibodies: anti-NG2 antibody (1:1000, Chemicon, USA), anti-CD11b (1:1000, Santa Cruz Biotechnology), anti-phospho-FAK (Tyr397, 1:500, Abcam), anti-FAK (1:1000, Abcam) or β-actin (1:2000, Abcam) overnight at 4 °C. Followed by HRP-conjugated secondary antibodies (Pierce) for 1 h at room temperature. After washing, the signals were detected using the SuperSignal West Dura extended duration substrate detection system (Thermo Scientific, Rockford, IL, USA) and X-ray exposure. The relative protein levels were determined after normalization with β-actin. Densitometric analysis of the bands was performed using the Quantity One software (Bio-Rad).

### ELISA

Blood samples were collected from WT and NG2 KO mice following FNA exposure. Serum was obtained by centrifugation of blood samples at 3000 r/min at 4 °C for 15 min. Cell culture supernatants were derived from transfected and non-transfected microglial cells after exposure to LPS. Nerve growth factor (NGF), glial derived neurotrophic factor (GDNF), tumor necrosis factor (TNF)-α and interleukin (IL)-1β levels in serum and cell culture supernatants were measured using commercial ELISA kits according to the manufacturer’s protocols (R&D Systems, Minneapolis, MN, USA). The ELISA kits used in the present study were designed against total target proteins and were not specific for mature or immature forms of the proteins.

### TUNEL assay

A TUNEL assay was performed on sections of the contralateral and ipsilateral FN collected on day 7 after FNA for the WT and KO mice using the *in situ* cell death detection kit according to the manufacturer’s protocol (Roche, Basel, Switzerland). Hematoxylin counterstaining of TUNEL sections allowed for the detection of nuclei. According to Gao *et al*.^40^, TUNEL-positive neuronal cells showing nuclear condensation/fragmentation and apoptotic bodies were considered as apoptotic cells. The number of apoptotic cells and the total number of cells were determined in three random microscopic fields (200X magnification). The apoptotic index was determined by expressing the number of apoptotic cells as a percentage of the total number of cells and values were averaged.

### Real-time PCR

Total RNA was extracted from ipsilateral FN or microglial cells using the RNeasy Mini kit (Qiagen) according to the manufacturer’s instructions. For reverse transcription, a 25 μL reaction mixture containing 2 μg of RNA, 2.5 μmol/L of oligo (dT) primer (Promega), and 200 U of Molony Murine Leukemia Virus Reverse Transcriptase (Promega) was incubated for 1 h at 42 °C. The reaction was stopped by heating for 10 min at 70 °C. Real time RT-PCR was then performed using a LightCycler kit (Roche Diagnostics, Indianapolis, IN, USA). Briefly, a reaction mixture containing 5 μL of 2 × SYBR Green I master mix (Qiagen), 0.5 μL of 10 μM of forward and reverse primer (for NG2: Forward, 5′-AATGAGGACCTGCTACACGG-3′, Reverse: 5′- CATCTGTAGTCAACAGCCGC-3′; for NGF: Forward, 5′-AACCAATAGCTGCCCGTGTG-3′, Reverse: 5′-ACACACTGAAAACTCCCCCA-3′; for GDNF: Forward, 5′-CGCTGACCAGTGACTCCAATA-3′, Reverse: 5′-GCGACCTTTCCCTCTGGAAT-3′; for TNF-α: Forward, 5′-ACGTCGTAGCAAACCACCAA-3′, Reverse: 5′-AAATGGCAAATCGGCTGACG-3′; for IL-1β: Forward, 5′-GCTACCTATGTCTTGCCCGT-3′, Reverse: 5′-GTCCATTGAGGTGGAGAGCTT-3′; for GAPDH: Forward, 5′-GTCGGTGTGAACGGATTTGG-3′, and Reverse: 5′-CCCCATTTGATGTTAGCGGG-3′) and 1.0 μL of cDNA, made up to a 10-μL final reaction volume, was used. The cycling conditions were as follows: 95 °C for 10 min; 40 cycles at 95 °C for 15 s and 60 °C for 30 s. The threshold cycle (Ct) was defined as the fractional cycle number at which the fluorescence passed the fixed threshold. The mRNA expression levels were quantified using the 2^−ΔΔCt^ method^41^, using the GAPDH mRNA expression level for normalization.

### Measurement of reactive oxygen species

2′,7′-dichloro-cluorescein diacetate (DCFH-DA) was used to detect intracellular reactive oxygen species (ROS) as previously described^42^. Cells were seeded in 6-well plates (3 × 10^6^ cells/well) and incubated for 24 h. ROS were detected using a Reactive Oxygen Species Assay Kit according to the manufacturer’s instructions (Beyotime institute of Biotechnology, China). Briefly, cells were harvested, and washed with PBS twice, then resuspended in serum-free media with 10 μM DCFH-DA added and incubated for 20 min at 37 °C in the dark. After a further three washes in PBS, FACSCalibur flow cytometry was performed (excitation wavelength: 485 nm, emission wavelength: 535 nm) to detect generation of the fluorescent-oxidized derivative of DCF. ROS generation was quantified by the median fluorescence intensity of 10,000 cells.

### Statistical analysis

The quantitative data are presented as the mean ± standard deviation. All data were statistically analyzed using the software GraphPad Prism 5 (GraphPad, San Diego, CA, USA). Differences between groups were evaluated using the Student’s t-test, and the significance among groups was determined using one-way ANOVA followed by Tukey’s post-hoc test. The level of significance was set at p < 0.05.

## Additional Information

**How to cite this article**: Zhu, L. *et al*. NG2 expression in microglial cells affects the expression of neurotrophic and proinflammatory factors by regulating FAK phosphorylation. *Sci. Rep.* **6**, 27983; doi: 10.1038/srep27983 (2016).

## Supplementary Material

Supplementary Information
