# Supplementary material for: NG2 expression in microglial cells affects the expression of neurotrophic and proinflammatory factors by regulating FAK phosphorylation
Source: Sci Rep. 2016 Jun 16;6:27983. doi: 10.1038/srep27983 (PMC4910048; doi:10.1038/srep27983)
Supplement: Supplementary Information [file srep27983-s1.pdf]

NG2 expression in microglial cells affects the expression of neurotrophic and proinflammatory factors by regulating FAK phosphorylation

Lie Zhu[1,#], Qing Su[2,#], Xiang Jie[1,#], Antang Liu[1], Hui Wang[1], Beiping He[3], Hua Jiang[1,]*

[1]Department of Plastic Surgery, Chang Zheng Hospital, Second Military Medical University, Shanghai 200003, China
[2]Department of Ophthalmology, Chang Zheng Hospital, Second Military Medical University, Shanghai 200003, China
[3]Department of Anatomy, Yong Loo Lin School of Medicine, National University of Singapore, Singapore

# Lie Zhu, Qing Su and Xiang Jie equally contributed to this work.

*Corresponding author:
Hua Jiang, Department of Plastic Surgery, Chang Zheng Hospital, Second Military Medical University, No. 415 Fengyang Road, Shanghai, China. 200003. E-mail: dosjh@126.com. Tel: 86-21-81885771; Fax: 86-21-63520020

SupplementaryMaterials and Methods

Behavioral tests

All behavioral tests were performed with the same cohort ofmice(8KO and 8 WT). Two tests were performed, arotarodtest and Catwalk analysis. For the rotarod test, an accelerating rotarod (Med Associates, Inc., St Albans, USA) was used to test neuromotor function as previously described[1]in the mice on three consecutive days. Initially a trial was performed of 2 min at 4 rpm, then four tests were performed with an acceleration of 4–40 rpm over 5 min. The delay before falling from the accelerating rod was measured and the results from the last test were recorded for each animal. ForCatwalk analysis, the Catwalk system (Noldus Information Technology, Wageningen, The Netherlands) was employed, as previously described[1,2], to assess gait. Briefly, each mouse had to cross a glass plate three times during which gait was monitored using a high speed camera. Stride length, duration of stance, swing speed andregularityindex (% regular patterns) were recorded and averaged from the triplicate measurements.

Clinical chemistry

WT and KO micewere orally administeredFAK inhibitor TAE226 (30 mg/kg) or methylcellulose as a vehiclecontrol once a day for 7 days.Clinical chemistry was performed on plasma samples collected from the miceon day 7 or 28 as previously described[3]. Briefly, mice were anesthetized with CO_2 and blood was collected via cardiac puncture exsanguination. The following parameters were evaluated: alanine aminotransferase, aspartate aminotransferase,alkaline phosphatase, albumin, urea, glucose, cholesterol, triglycerides, total bilirubin and total protein, using anautomatic chemistry analyzer (Analyst III,Hemagen Diagnostics, Inc., Columbia, MD, USA).

SupplementaryReferences

1 Verheijden, S. *et al.*Peroxisomal multifunctional protein-2 deficiency causes neuroinflammation and degeneration of Purkinje cells independent of very long chain fatty acid accumulation. *Neurobiology of disease***58**, 258-269, (2013).

2 Vandeputte, C. *et al.* Automated quantitative gait analysis in animal models of movement disorders. *BMC neuroscience***11**, 92, (2010).

3 Golubovskaya, V., Curtin, L., Groman, A., Sexton, S. &Cance, W. G. In vivo toxicity, metabolism and pharmacokinetic properties of FAK inhibitor 14 or Y15 (1, 2, 4, 5-benzenetetramine tetrahydrochloride). *Archives of toxicology***89**, 1095-1101, (2015).

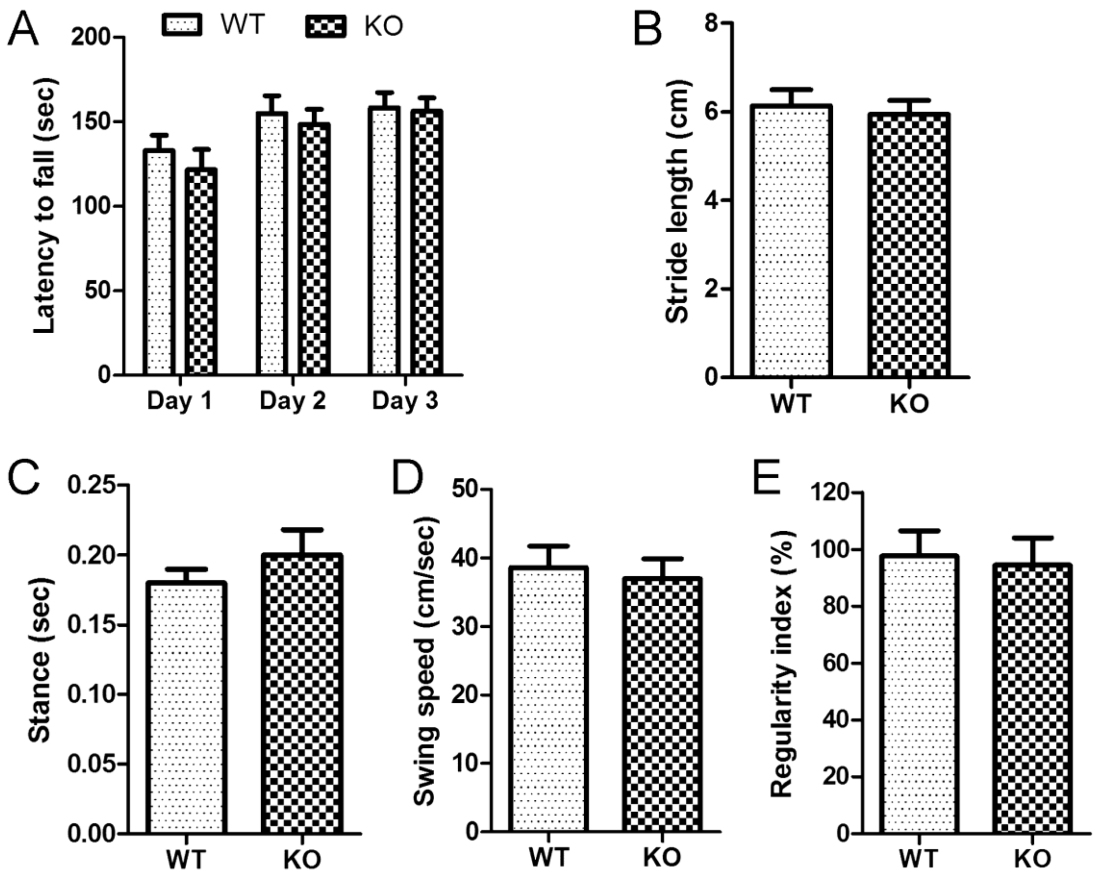

Fig. S1 Rotarod andCatwalk analyses to test the motor faculties of the WT and KO mice. (A) Rotarod tests were performed on three consecutive days, and the latencytime (sec) before falling from the accelerating rod was measured for each mouse. (B–E) Catwalk tests were performed to analyze gait by measuring the following parameters: (B) stride length, (C) duration of stance, (D) swing speed, and (E) regularityindex (% regular patterns).

Table S1.Analysis of clinical chemistry parametersinWT and KO mice treated with the TAE226 FAK inhibitor or the vehicle only control

Analyte	WT mice		KO mice	
	Control	TAE226	Control	TAE226
ALT (U/L)	41.78±6.81	44.35±4.95	40.98±7.05	44.82±5.16
AST (U/L)	115.07±14.46	117.92±21.07	114.62±20.18	120.94±25.37
ALP (U/L)	135.62±21.19	147.65±28.54	129.41±25.24	136.32±15.03
Albumin (g/dL)	2.85±0.22	3.04±0.38	2.73±0.27	3.31±0.21
Urea (mg/dL)	18.47±3.57	25.71±2.45	16.51±3.08	22.47±1.89
Glucose (mg/dL)	221.38±25.14	217.57±37.51	224.92±40.25	230.53±23.22
Cholesterol (mg/dL)	156.54±17.27	158.16±21.33	149.35±20.14	150.40±17.05
Triglycerides (mg/dL)	133.31±20.08	131.80±15.47	134.84±25.11	138.17±21.24
Total bilirubin (mg/dL)	0.18±0.07	0.23±0.05	0.20±0.01	0.22±0.08
Total protein (g/dL)	4.85±0.23	4.39±0.51	4.48±0.72	4.74±0.16

Values are expressed as the mean±standard deviation. n = 6 mice per group.ALT: alanine aminotransferase; AST: aspartate aminotransferase; ALP: alkaline phosphatase.
